# Regional Heterogeneity of Perivascular Adipose Tissue: Morphology, Origin, and Secretome

**DOI:** 10.3389/fphar.2021.697720

**Published:** 2021-06-22

**Authors:** Xinzhi Li, Zhongyuan Ma, Yi Zhun Zhu

**Affiliations:** ^1^ School of Pharmacy and State Key Laboratory of Quality Research in Chinese Medicine, Macau University of Science and Technology, Macau, China; ^2^ Department of Cardiothoracic Surgery, Zhuhai People’s Hospital, Jinan University Medical School, Guangzhou, China

**Keywords:** adipocyte, preadipocyte, adipokine, cellular heterogeneity, obesity, vascular inflammation

## Abstract

Perivascular adipose tissue (PVAT) is a unique fat depot with local and systemic impacts. PVATs are anatomically, developmentally, and functionally different from classical adipose tissues and they are also different from each other. PVAT adipocytes originate from different progenitors and precursors. They can produce and secrete a wide range of autocrine and paracrine factors, many of which are vasoactive modulators. In the context of obesity-associated low-grade inflammation, these phenotypic and functional differences become more evident. In this review, we focus on the recent findings of PVAT’s heterogeneity by comparing commonly studied adipose tissues around the thoracic aorta (tPVAT), abdominal aorta (aPVAT), and mesenteric artery (mPVAT). Distinct origins and developmental trajectory of PVAT adipocyte potentially contribute to regional heterogeneity. Regional differences also exist in ways how PVAT communicates with its neighboring vasculature by producing specific adipokines, vascular tone regulators, and extracellular vesicles in a given microenvironment. These insights may inspire new therapeutic strategies targeting the PVAT.

## Introduction

Obesity is becoming a substantial public health concern since it gives rise to a wide range of disorders (Olshansky et al., 2005). Obesity prevalence has tripled since 1975 and it is now one of the WHO’s priorities to cease the quick rise in obesity (Jaacks et al., 2019). The burgeoning global epidemic of obesity, consequently leading to type 2 diabetes, dyslipidemias, cardiovascular disease, and even some cancers will soon be devastating if actions are not taken (Alberti et al., 2009; Grundy, 2012; Scheen and Van Gaal, 2014). Obesity occurs due to excess energy intake, dramatic fat mass accumulation caused by increased adipocyte size (hypertrophy), or increased adipocyte number (hyperplasia), or both. Given the systemic impacts, obesity cannot be seen simply as a fat accumulation, and it goes hand-in-hand with many metabolic complications. People often experienced obesity as a chronic affliction characterized by low-grade inflammation. Despite the absence of the four cardinal signs of typical inflammation, the unresolved inflammation, entwined with fibrosis and defected angiogenesis-induced hypoxia, is a common pathway in the development of various cardiometabolic diseases.

Excessive fat accumulation can happen in various adipose depots, including a kind of unique adipose tissue known as perivascular adipose tissue (PVAT). PVAT surrounds most blood vessels except capillaries, pulmonary and cerebral blood vessels. PVAT plays wide-ranging physiological roles far beyond supporting connective tissue. It is now considered a metabolically active organ that regulates vascular function in both autocrine and paracrine fashions by producing various adipokines (Rajsheker et al., 2010; Van de Voorde et al., 2013). Over the decades, our understanding of PVAT biology has increased simultaneously with a rising prevalence of obesity and related metabolic complications. Not only does PVAT actively maintain vascular homeostasis, but it also markedly modulates various inflammation-related cardiovascular diseases. Due to the proximity of PVAT to the associated blood vessels, PAVT can be a unique target to improve arterial function in the setting of obesity.

PVATs are not only different from classical adipose tissues, they vary from location to location developmentally and functionally (Chang et al., 2020). Most intriguingly, PVAT’s origin largely remains unknown, despite the development of cellular fate mapping and lineage tracing in other fat depots (Sanchez-Gurmaches et al., 2016; Guimaraes-Camboa et al., 2017; Schwalie et al., 2018; Merrick et al., 2019; Shao et al., 2019; Cattaneo et al., 2020). Numerous publications have presented us with a bulk of mixed information, given the fact that these publications always include more than one PVAT, which challenges readers outside of the field. The general information on the pathophysiological functions of PVAT has been the subject of many recent comprehensive reviews (Nosalski and Guzik, 2017; Hildebrand et al., 2018; Chang et al., 2020). In this review, we aim to form a conceptual picture of PVAT heterogeneity, mainly focusing on histology, developmental origin, and briefly on secretome.

## Depot-Specific and Polychromatic Adipose Tissue

### Nomenclature of Perivascular Adipose Tissues

**FIGURE 1:**
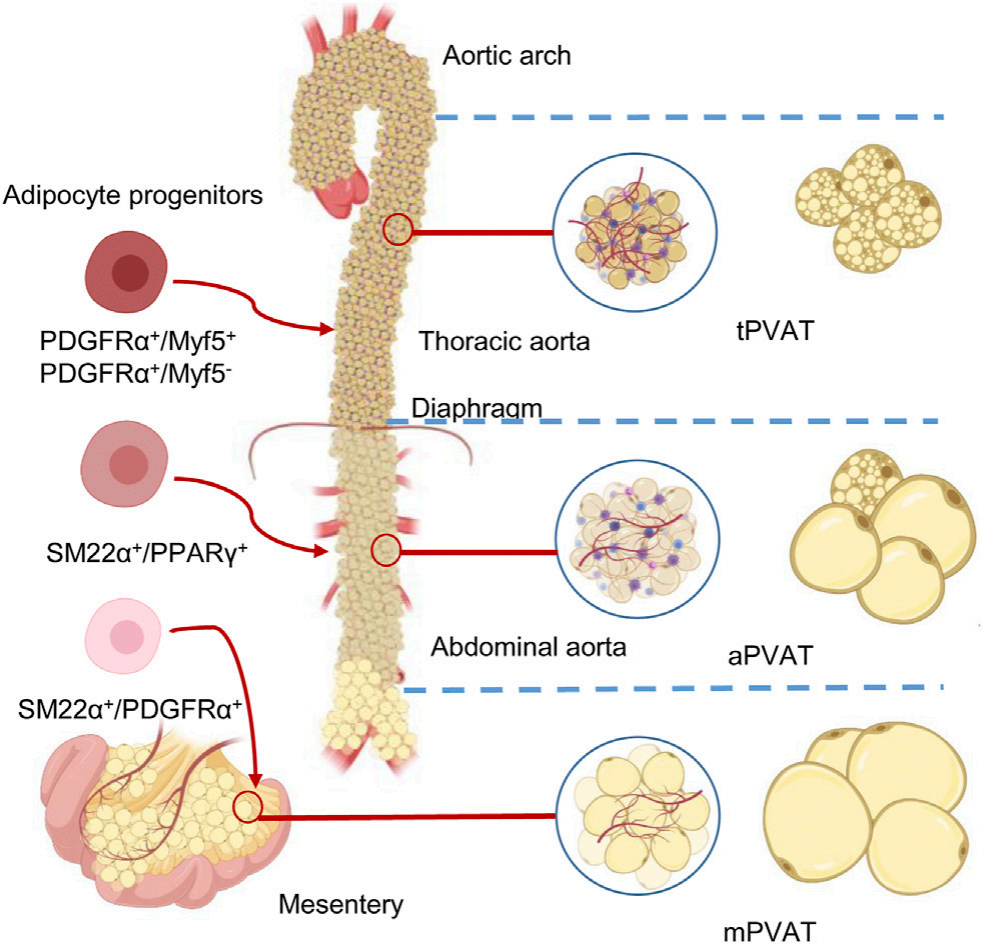
Anatomical, morphological, and developmental differences of the perivascular adipose tissues (PVATs). The color differences reflect the different proportions of brown adipocytes in the adipose tissue. Adipose tissues around thoracic aorta (tPVAT), abdominal aorta (aPVAT), and mesenteric artery (mPVAT) have distinct fat cell populations that are derived from different adipocyte progenitors. These progenitors could be identified by highly expressed cell markers, such as platelet-derived growth factor receptor-α (PGDFRα), myogenic factor 5 (Myf5), smooth muscle protein 22-α (SM22α), and peroxisome proliferator-activated receptor-γ (PPARγ).

A PVAT is generally named after its adjacent vessel’s name, such as pericoronary adipose tissue, referring to the fat tissue around the coronary artery. Over the years, various non-standard names were used for the same PVAT, which can be merely confusing or even misleading. For instance, “cardiac” PVAT surrounding the coronary artery or epicardial adipose tissue was abbreviated as either “C-PVAT” (Drosos et al., 2016), or “PVAT-CA” (Lu et al., 2017), or even “epi” for shorter (Mazurek et al., 2003). It is not uncommon that these names are mistakenly referred to, for example, in the case of pericardial and epicardial fat (Iacobellis, 2009). These facts reason the need to standardize nomenclature for PVATs. Some research articles have used a combined format to define these concepts (Tran et al., 2018; Ye et al., 2019). Throughout this review, we will use their traditional names according to their locations but with some specifications. In particular, we “standardized” these names by introducing a common name, “PVAT,” following a lowercase letter, indicating where it is localized. For example, pericoronary adipose tissue is termed as “cPVAT,” thoracic periaortic adipose tissue is abbreviated as “tPVAT,” and abdominal periaortic adipose tissue as “aPVAT.” Adipose tissue around the mesenteric artery is called “mPVAT.” A comparison among the four most commonly studied PVATs is presented in Table 1 and Figure 1.

**TABLE 1:** Reginal differences of perivascular adipose tissue.

	cPVAT	tPVAT	aPVAT	mPVAT
Anatomical location	Pericoronary, adjacent to the heart	Thoracic periaortic, from aortic arch at T4 to the T10–T11 vertebrae, above the diaphragm	Abdominal periaortic, from below the diaphragm to femoral bifurcation	Mesenteric arterial, around the resistance mesenteric arteries
Other name/acronym	Epicardial adipose tissue (EAT), C-PVAT, PVAT-CA	Thoracic PVAT, Thor PVAT	Abd PVAT	Adipose tissue of mesenteric bed
Predominant adipocyte	Beige Sacks et al. (2013)	Brown	White, very few beige Police et al. (2009)	White
Morphology of adipocyte	Spotted multilocular, but mainly small unilocular adipocytes	Multilocular brown adipocytes, abundant mitochondria Fitzgibbons et al. (2011), Padilla et al. (2013)	Primarily unilocular, fewer mitochondria Police et al. (2009), Padilla et al. (2013)	Large unilocular Galvez et al. (2006), Gil-Ortega et al. (2010)
Highly expressed gene	UCP-1, PRDM16, PGC-1α, PPARγ, and the beige adipocyte-specific marker CD137 Sacks et al. (2009), Sacks et al. (2013)	UCP-1, PRDM16, PGC-1α Tran et al. (2018); Cidea, PPARγ Fitzgibbons et al. (2011); EBF2 Angueira et al. (2021)	Hoxc8, Nnat, Sncg, Mest Tran et al. (2018)	Hoxc8, Tcf21, Tbx1, Pat2, dermatopontin Walden et al. (2012), Friederich-Persson et al. (2017)
Developmental origin	Splanchnic mesoderm Sacks et al. (2013)	Multiple lineages including ectoderm-derived neural crest (periaoritic arch) and mesoderm Ye et al. (2019)	Mesoderm	Mesothelial lineage
Progenitor/stem cell marker	Unknown	Pparg-dependent Xiong et al. (2018); Myf5^+^/Myf5^−^ (Ye et al. (2019); Pdgfra^+^	Pparg-dependent, SM22α^+^ Chang et al. (2012)	CD34, CD44, and Pdgfra^+^ Contreras et al. (2016); SM22α^+^ Chang et al. (2012)
Pro-/anti-inflammatory	Pro-inflammation and pro-atherosclerosis Numaguchi et al. (2019)	Anti-atherosclerosis and anti-inflammation in mouse; Fitzgibbons et al. (2011); proatherosclerosis in human Lehman et al. (2010), Britton et al. (2012)	Pro-inflammation and prone to aneurysm formation Police et al. (2009)	Pro-inflammation and Pro-atherosclerosis Sena et al. (2017)

### Multicolored Adipose Tissue

Adipose tissues are empirically color-coded as white (WAT), brown (BAT), and beige/brown-in-white (brite) adipose tissues. The color explicitly reflects the main adipocyte population and the amount of iron-containing mitochondria in each adipocyte. The more mitochondria, the darker the color is. In addition to major depots, there are other location-specific adipose tissues, including mammary “pink” adipose tissue and bone marrow adipose tissue, which is primarily red in young individuals and turns yellow during aging. It would undoubtedly be oversimplified if we classify adipose tissues based solely on the appearing color and neglect their complexity and heterogeneity. Classical inter-scapular BAT has been previously assumed to contain a homogeneous population of brown adipocytes. A recent study has revealed a new brown adipocyte subpopulation with low thermogenic activity coexists with the classical high-thermogenic brown adipocytes (Song et al., 2020). More interestingly, these two brown adipocyte subpopulations may interconvert dynamically in response to the environmental temperature (Song et al., 2020). Using AdipoChaser mouse models, Wang et al. also demonstrated that “pink” mammary adipose tissue can undergo reversible dedifferentiation during lactation, where de-differentiated fibroblasts proliferate and re-differentiate into adipocytes upon weaning (Wang et al., 2018).

PVAT’s color is not visually presented. Instead, it is determined by the subcellular composition and enrichment of specific genetic biomarkers. For example, tPVAT, characterized as brown adipose tissue, is labeled with highly expressed uncoupling protein-1 (UCP-1) and cell death activator CIDE-A (Cidea) (Tran et al., 2018). Conversely, aPVAT is seemingly white but contains a mixture of beige and white adipocytes (Padilla et al., 2013). Therefore, when we talk about the color of a PVAT, we mainly use generalized terminology to reflect their cellular characteristics and corresponding genetic profiles. Generally speaking, white PVAT adipocytes share a lot of similarities with their visceral counterparts, which are morphologically classified by the appearance of large unilocular lipid droplets, fewer mitochondria, and small cytoplasmic volumes. Brown PVAT adipocytes, on the other hand, are distinguished by multilocular lipid droplets and high density of mitochondria. In rodents, arteries including the mesenteric, carotid, and femoral are surrounded by WAT, while the thoracic aorta is encircled by BAT and the abdominal aorta by beige PVAT (a mixture of white and brown adipocytes) (Brown et al., 2014).

## Histological Distinctions of Perivascular Adipose Tissues

Although the majority of adipose tissue, either by volume or by function, is adipocytes, white adipocytes comprise only one-third of the total cell number in WAT. The remaining 70% of cells are a heterogeneous collection of largely undefined stromal and immune cells (Sarantopoulos et al., 2018). Understandably, PVAT is composed of adipocytes, nerves, and a stromal vascular fraction consisting of monocytes, endothelial cells, pericytes, macrophages, T cells, and mesenchymal stem cells (Chatterjee et al., 2009). Large vessels are separated from their PVATs by a layer of adventitia composed of elastic bundles, fibroblasts, nerves, and vasa vasorum. On the other hand, PVATs around small vessels and microvessels are seen as a seamless continuum from their associated blood vessels. Different PVATs are dominated by diverse adipocytes, indicating regional phenotypic heterogeneity in a given vasculature (Fitzgibbons et al., 2011).

### Histology of Pericoronary Adipose Tissue

Broadly, cardiac fat depots include epicardial and pericardial adipose tissues. A big portion of epicardial fat surrounding the coronary arteries is called pericoronary adipose tissue (cPVAT) (Fernandez-Alfonso et al., 2017). Human cPVAT was firstly considered as a unique subtype of WAT since the cellular morphology and gene profile were more consistent with WAT than BAT (Chatterjee et al., 2009). Differentiated pericoronary adipocytes are irregularly shaped, smaller in size, and lower in lipid accumulation relative to subcutaneous and perirenal counterparts (Chatterjee et al., 2009). Meanwhile, human cPVAT exhibits an increased capacity to attract immune cells and induce angiogenesis, contributing to coronary atherosclerosis development (Chatterjee et al., 2009). Notably, these features were observed in differentiated preadipocytes *in vitro*, which might differ from *in vivo* physiological settings.

Evidence indeed showed cPVAT was composed of small unilocular adipocytes positively stained with brown fat marker UCP-1 (Chatterjee et al., 2013; Sacks et al., 2013). Sacks et al. further proposed that it would be more reasonable that cPVAT might closely resemble beige adipocytes (Sacks et al., 2013; Sacks and Symonds, 2013). Consistent expression of UCP-1 in cPVAT has been repeatedly reported since then (Chechi et al., 2017), but less expression of adiponectin, an anti-inflammatory adipokine was also reported (Numaguchi et al., 2019). Thus cPVAT can be classified as beige adipose tissue, with a large proportion of white adipocytes, which may contribute to coronary atherosclerosis development.

### Histology of Thoracic Periaortic Adipose Tissue

**FIGURE 2:**
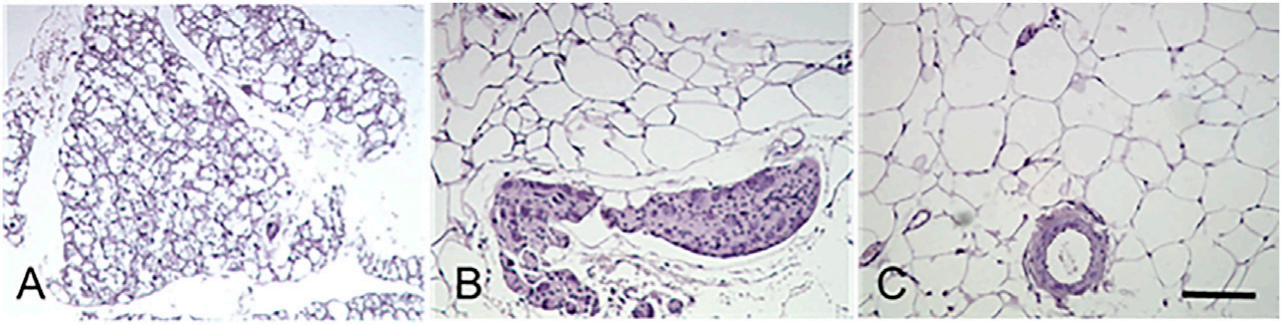
Histological characteristics of the perivascular adipose tissues. Murine adipose tissues around thoracic aorta (tPVAT, panel **(A)**), abdominal aorta (aPVAT, panel **(B)**), and mesenteric artery (mPVAT, panel **(C)**) were stained by hematoxylin and eosin. Brown adipocytes in tPVAT are characterized by small-sized, multilocular lipid droplets, whereas white adipocytes in mPVAT are classified by the appearance of a larger unilocular lipid droplet and small cytoplasmic space. aPVAT has mixed adipocyte populations. Scale bar, 100 µm.

Thoracic periaortic PVAT (tPVAT) expands from the aortic arch at the T4 vertebra to the diaphragm’s aortic hiatus at the T10–T11 vertebrae. The comparison of adipocyte size, inflammation, and macrophage polarization indicated that tPVAT was close to subcutaneous adipose tissue (Fitzgibbons et al., 2011). It is now widely accepted that tPVAT is morphologically and functionally like BAT (Figure 2A). Multilocular brown adipocytes and abundant mitochondria were observed under light or electron microscopy (Fitzgibbons et al., 2011; Padilla et al., 2013), which might contribute to the decreased atherosclerotic plaque burden (Fitzgibbons et al., 2011).

In tPVAT, the adipocyte phenotypic switching via specific signaling pathways is evident in both whitening and browning directions. In a diet-induced obesity mouse model, tPVAT adipocytes become “whiter”, with a primarily unilocular appearance and larger lipid (Galvez-Prieto et al., 2008; Sacks and Symonds, 2013). Aging together with miR-146b-3p downregulation could decrease browning (Pan et al., 2018). Reduced brown adipogenic differentiation of resident stromal cells via loss of peroxisome proliferator-activated receptor-γ (PPARγ) coactivator-1 α (PGC-1α) was also observed in aged animals (Pan et al., 2019). Cold is a capable inducer for perivascular adipocyte to re-brown and to restore protective effects on metabolism and vascular function (Boa et al., 2017; Bussey et al., 2018). This relationship between tPVAT and adiposity, employed as a predictor for cardiovascular diseases, warrants further investigation in a clinical setting.

### Histology of Abdominal Periaortic Adipose Tissue

Abdominal periaortic adipose tissue (aPVAT) is the adipose segments surrounding the abdominal aorta from below the diaphragm to the femoral bifurcation. As a continuum of thoracic aorta, the abdominal aorta is encircled by 4- to 10-fold more adipose tissue than the adipose tissue around the aortic arch in rats (Henrichot et al., 2005). aPVAT is more prone to accumulation when exposed to a high-fat diet and shows significant adipocyte hyperplasia and hypertrophy (Henrichot et al., 2005). A comparison with tPVAT demonstrates that aPVAT contains a mixture of cells, with a predominant proportion of unilocular white adipocytes and a small portion of brown adipocytes (Police et al., 2009). Moreover, the average aPVAT adipocyte size is much bigger than that of tPVAT (Figure 2B). Electron microscopy demonstrated that aPVAT was composed of white adipocytes with fewer mitochondria (Padilla et al., 2013). Upon a 4 month high-fat diet feeding, mouse aPVAT showed higher gene expression of inflammatory cytokines than tPVAT. Leptin coincided with the inflammatory cytokines in aPAVT, whereas UCP-1 was elevated in tPVAT but suppressed in aPVAT throughout diet-induced obesity (Police et al., 2009). Further studies provided evidence that only aPVAT-, but not tPVAT-conditioned medium could promote inflammatory cytokine MCP-1 generation and macrophage migration (Police et al., 2009). aPVAT was found to express a higher level of inflammatory receptors, including CD11c, IL-6R, and TNFR1/2 relative to tPVAT and inter-scapular brown adipose tissues (Padilla et al., 2013). These convincing pieces of evidence differentiate two segments of PVAT, although they wrap along the same artery. These intriguing features remain enigmatic due to the lack of solid advances in the developmental origin, transdifferentiation, and cellular fate mapping of PVAT.

### Histology of Mesenteric Perivascular Adipose Tissue

In humans and mice, the fat tissue around the resistance mesenteric arteries forms mesenteric perivascular adipose tissue (mPVAT) (Figure 2C). Among adipose tissues collected from different locations, mPVAT was traditionally categorized as visceral WAT (Walden et al., 2012) with slightly smaller adipocyte size than classical visceral adipocytes (Caesar et al., 2010). However, mPVAT adipocytes are four times larger than periaortic adipocytes, and mPVAT expresses much less brown adipocyte marker of UCP-1 and PR domaining containing 16 (PRDM16) compared with brown adipocytes (Galvez-Prieto et al., 2008; Walden et al., 2012). The enlarged cross-sectional area of mPVAT adipocytes during obesity could be reduced but not completely reversed after caloric restriction (Bussey et al., 2016).

mPVAT expressed WAT specific marker transcription factor 21 (Tcf21), beige specific marker T-box protein 1 (Tbx1), and transcription factor Pat2. Besides, treatment with a β3-agonist, CL-316,243, could increase beige markers in mPVAT (Friederich-Persson et al., 2017). Other brown adipocyte gene markers, including myosin regulatory light chain (Mylpf), lim homebox 8 (Lhx8), zinc fingers in the cerebellum1 (Zic1), and T-box 15 (Tbx15) are barely seen in mPVAT, whereas a series of white adipose genes such as homeobox C8 (Hoxc8), transcription factor 21 (Tcf21), and dermatopontin (Dpt) are highly expressed in mPVAT (Walden et al., 2012). These findings adequately help classify mPVAT as a WAT.

## Different Origin of Perivascular Adipose Tissues

Typical mature adipocytes originate from progenitor cells, which are committed preadipocytes derived from stem cells of multiple sources (Rodeheffer et al., 2008). The distinction between WAT and BAT reasonably leads to a simple classification of two precursor populations, giving rise to white and brown adipocytes, respectively. A commonly known classifier is *myf5*, which encodes myogenic factor 5 (Myf5) (Timmons et al., 2007), whereas PRDM16 may be a controller for brown adipocyte generation (Seale et al., 2008). However, using mTmG reporter, a study revealed that white adipocytes in the subcutaneous and retroperitoneal WAT also originate from Myf5-expressing precursors. This approach also demonstrated that Myf5 expression could not track many brown adipocytes (Sanchez-Gurmaches and Guertin, 2014a). The adipogenic capacity of vasculature-residing mural cells (e.g., pericytes) in the adipose tissue was well documented in many papers (Tang et al., 2008; Gupta et al., 2012; Tran et al., 2012; Berry and Rodeheffer, 2013). This statement is challenged by a study where pericytes do not contribute to adipocytes’ generation, although they seem to act as progenitors *in vitro* (Guimaraes-Camboa et al., 2017). Merrick et al. further indicated that reticular interstitium rather than the vasculature is the residing site for interstitial progenitor cells, which give rise to the preadipocytes expressing intercellular adhesion molecule–1 and another group of cells expressing protein CD142 (Merrick et al., 2019). Most recent results further revealed that only fibroblasts, neither mural nor endothelial cells, are cells of the vascular wall with significant adipogenic potential *in vivo* in both WAT and BAT (Cattaneo et al., 2020). Thus developmental origins of adipose tissue and the mechanisms controlling its expansion are just beginning and more intriguing findings are expected to come soon. Adipocytes of different PVATs may originate from distinct precursors (Hepler et al., 2017; Tran et al., 2018). Despite the aforementioned development of cellular fate mapping and lineage tracing in other adipose depots, the origins of PVAT adipocytes, in general, are barely known. This session presents distinct adipocyte development in Table 1 and Figure 1. A clear definition of adipocyte origin can reveal the determined precursors and the regulatory mechanisms.

### Origin of Epicardial and Periaortic Arch Perivascular Adipose Tissue

Epicardial fat originates from the splanchnic mesoderm in human (Sacks et al., 2013) and in mouse (Walden et al., 2012). Periaortic adipose tissue is potentially derived from Myf5^+^ progenitors (Sanchez-Gurmaches and Guertin, 2014a). A more recent study showed that periaortic adipocyte progenitors expressed smooth muscle protein 22-alpha (SM22α) during development. Besides, knockout of PPARγ in neural crest cells leads to developmental delay of the periaortic arch PVAT (Fu et al., 2019). This evidence indicates that periaortic arch PVAT adipocytes have multiple lineages, mainly from ectoderm-derived neural crest cells, rather than the mesoderm-derived Myf5^+^ progenitors (Fu et al., 2019). A possible explanation is that ectoderm-derived neural crest cells have a broad differentiation potential and give rise to a diverse range of cell types. For example, neural crest cells were once identified as one of the progenitors of white adipocytes (Sanchez-Gurmaches and Guertin, 2014b). Another study revealed that neural crest-derived cells resided along the vessels within the subcutaneous adipose tissue. These results demonstrate that neural crest-derived adipocyte-committed progenitors contribute to adipogenesis (Sowa et al., 2013).

### Origin of Thoracic Periaortic Adipose Tissue

Unlike periaortic arch PVAT, neural crest cells do not contribute to tPVAT development (Ye et al., 2019). Only about 10–30% of the brown adipocytes in tPVAT derive from Myf5^+^ sources (Ye et al., 2019). A lineage-tracing study further elaborates that anterior tPVAT adipocytes can be traced to SM22α^+^ progenitors, whereas left lateral tPVAT presents both SM22α^+^ and Myf5^+^ features (Ye et al., 2019). However, recent cell differentiation assays and genetic fate mapping studies show that fibroblastic progenitor cells but not vascular smooth muscle cells (VSMCs) are responsible for tPVAT adipogenesis (Angueira et al., 2021), which contradicts the previous findings (Chang et al., 2012; Ye et al., 2019). Progenitor cells for tPVAT are from a fibroblastic lineage, including (Pdgfra^+^; Ly6a^+^; Pparg^−^) and preadipocytes (Pdgfra^+^; Ly6a^−^; Pparg^+^). Bona fide VSMCs were not found to contribute to adipocyte formation in tPVAT (Angueira et al., 2021). Single-cell transcriptomic analyses both at embryonic (E18) and perinatal (P3, after birth) stages identified transcription factor early B cell factor-2 (EBF2) as a critical modulator of BAT (Angueira et al., 2020) and tPVAT development (Angueira et al., 2021).

### Origin of Abdominal Periaortic Adipose Tissue

Abdominal PVAT preadipocytes demonstrate lower brown adipocyte developmental transcription factors relative to tPVAT (Tran et al., 2018). Abdominal PVAT lacks *Zic1* gene, encoding zinc finger proteins that are critical for early BAT development (Contreras et al., 2016). In the absence of the adipogenic transcription factor PPARγ in the VSMCs, failure of aPVAT development was observed (Chang et al., 2012). These findings demonstrate that aPVAT shares, at least to some extent, similar developmental origins with SM22α^+^ and PPARγ^+^ VSMCs.

### Origin of Mesenteric Perivascular Adipose Tissue

Few studies have focused on the developmental origin of mPVAT, and some comparative data may provide limited clues. Given the proximity, mPVAT’s developmental origins are thought to be similar to the visceral adipose tissue. Indeed, both mPVAT and perigonadal adipose tissue expressed comparable levels of white adipocyte signature gene *Tcf21* and the brite adipocyte-specific genes *Tbx1* and *Tmem26* (Contreras et al., 2016). Mesenteric and perigonadal adipose tissue are also found to share the same mesothelial origin in lineage tracing experiments, and preadipocytes of mPVAT have a transcriptional profile closer to that of subcutaneous, but not omental preadipocytes (Chau et al., 2014). Also, in this cell lineage analysis, 28% of mature mesenteric adipocytes were Wt1+ positive, suggesting the source of mesenchymal stem cells in mesenteric adipocytes is different from that of BAT, where Wt1 expression is undetectable (Chau et al., 2014). Chang et al. have suggested that mPVAT shares a developmental origin with VSMCs because the deletion of the PPAR γ in VSMCs resulted in a dramatic loss of mPVAT (Chang et al., 2012).

## Different Secretome of Perivascular Adipose Tissues

Adipose tissue is capable to synthesize and secrete various substances just as endocrine cells do. In this sense, adipose tissue is the largest endocrine organ, and more than 600 hundred identified factors produced by adipocytes are collectively termed adipokines or adipocytokines (Halberg et al., 2008; Lehr et al., 2012). Paracrine crosstalk between PVAT and its neighboring artery, also known as “vasocrine” communication, actively regulates vascular inflammation and arterial remodeling (Yudkin et al., 2005). Anatomically distinct PVAT depots can release a quite different range of adipokines. Previous literatures have deeply explored and frequently revisited this topic (Omar et al., 2014; Owen et al., 2014; Akoumianakis et al., 2017; Nosalski and Guzik, 2017; Xia and Li, 2017; Oikonomou and Antoniades, 2019; Chang et al., 2020; Kim et al., 2020). We briefly summarized PVAT-derived adipokines, vascular tone regulators, and newly discovered exosomes/extracellular vesicles in this part. Unlike previous reviews, these factors are categorized by specific PVAT depots where detectable levels are reported (e.g. PCR, Western-blot, or immunostaining).

### Pro- and Anti-Inflammatory Adipokines of Perivascular Adipose Tissues

Most PVAT-generated cytokines/chemokines such as tumor necrosis factor (TNF)-α, interleukin-6 (IL-6), plasminogen activator inhibitor-1 (PAI-1), and monocyte chemoattractant protein-1 (MCP-1), are pro-inflammatory and pro-atherosclerotic. Adiponectin is one of the few anti-inflammatory factors that possess multiple salutary effects for cardiovascular disease prevention (Xu and Vanhoutte, 2012). Phenotypic differences between tPVAT and aPVAT are evident that tPVAT generates much less pro-inflammatory cytokines, and is thus resistant to diet-induced inflammation (Police et al., 2009; Fitzgibbons et al., 2011). mPVAT and aPVAT are similarly prone to the expansion of adipocytes and diet-induced inflammation (Li et al., 2019). However, mPVAT is more sensitive to the high-fat diet challenge, where the adipose “browning” genes are dramatically down-regulated (Hou et al., 2016). Previous reviews in this field have elaborated on PVAT-derived adipokines and their interactions with other vascular cells (Brown et al., 2014; Gil-Ortega et al., 2015; Akoumianakis et al., 2017; Kim et al., 2019; Chang et al., 2020). The PVAT-generated representative pro- and anti-inflammatory factors are briefly updated in Table 2. Note that one type of adipokine can be generated from multiple sites and at the same time one PVAT can release a variety of adipokines. Members of the same adipokine family (i.e., pro-inflammatory or constricting) all share the same predicted vascular function.

**TABLE 2:** PVAT-generated autocrine and paracrine factors.

	Anti-inflammatory factors	Pro-inflammatory factors
cPVAT	Adiponectin Iacobellis et al. (2005), Cheng et al. (2008), Chatterjee et al. (2009), Spiroglou et al. (2010)	MCP-1 (CCL2), IL-8, IL-6, Leptin, MIP-1α (CCL3) Chatterjee et al. (2009)
	Omentin Gaborit et al. (2015)	TNF-α, IL-1β Mazurek et al. (2003)
	IL-10 Gruzdeva et al. (2019), Numaguchi et al. (2019)	IL-13 Vianello et al. (2019)
		Visfatin, TNF-α Cheng et al. (2008)
		Chemerin, Vispin Spiroglou et al. (2010)
		Apelin Toczylowski et al. (2019)
		Plasminogen activator inhibitor-1, Resistin Langheim et al. (2010)
tPVAT	Adiponectin Chatterjee et al. (2009)	IL-6, TNF-α, RANTES (CCL5), MCP-1 (CCL2) Manka et al. (2014), Xiong et al. (2018)
	IL-10, IL-4 Dobrian et al. (2015)	IL-17A Smith et al. (2010)
		IL12p40, CXCL10, CX3CL1, CCL2, CXCL16 Dobrian et al. (2015)
		Leptin Chatterjee et al. (2009)
		Resistin Jung et al. (2006)
		Visfatin Wang et al. (2009)
aPVAT	Adiponectin Kostopoulos et al. (2014), Horimatsu et al. (2018)	IL-1, IL-6 Lohmann et al. (2009)
	IL-10 (Sakamoto et al. (2014)	MIP-1α (CCL3) Moos et al. (2005)
		RANTES Sakamoto et al. (2014)
		IL-8, MCP-1Henrichot et al. (2005)
		Leptin Police et al. (2009)
		Platelet-derived growth factor-D Zhang et al. (2018)
		Resistin and visfatin Spiroglou et al. (2010), Park et al. (2014)
		Chemerin Spiroglou et al. 2010)
White PVAT (mesenteric, femoral, common carotid)	Adiponectin Schmid et al. (2011), Weston et al. (2013)	IFN-γ, IL-17 Smith et al. (2010)
	IL-10 Kassan et al. (2011)	MCP-1, TNF-α, IL-6, Plasminogen activator inhibitor-1 Takaoka et al. (2010)
		CCL2, CCL5 and CX3CL1, IL-1β, MIP-1α Lohmann et al. (2009)

CX3CL1, C-X3-C motif chemokine ligand 1; CXCL10, C-X-C motif chemokine ligand 10; CXCL16, C-X-C motif chemokine ligand 16; IFN-γ, Interferon-γ; IL, interleukin; MCP-1 (CCL2), monocyte chemoattractant protein-1 (C-C motif chemokine ligand 2); MIP-1α (CCL3), macrophage inflammatory protein-1α (C-C motif chemokine ligand 3); RANTES (CCL5), Regulated upon activation, normal T cell expressed and presumably secreted (C-C motif chemokine ligand 5); TNF-α, Tumor necrosis factor-α.

### Perivascular Adipose Tissue-Derived Vasodilators and Contracting Factors

Many PVAT-derived factors are also vascular tone regulators. Since the discovery of adipocyte-derived relaxing factor (ADRF) in 2002 (Lohn et al., 2002), understanding of the mechanisms by which PVAT maintains vascular homeostasis has been sought. A wide range of PVAT-derived relaxing factors have been proposed, such as adiponectin (Lynch et al., 2013), prostacyclin (Chang et al., 2012), gaseous molecules (e.g. NO and H_2_S) (Szasz and Webb, 2012), methyl palmitate (Lee et al., 2011), angiotensin 1–7 (Lee et al., 2009), and omentin (Yamawaki et al., 2010). Potential contracting candidates include angiotensin II (Lu et al., 2010), endothelin-1 (Almabrouk et al., 2014; Tano et al., 2014), and resistin (Small et al., 2019) released by adipocytes. Depending on PVAT location and different circumstances, some factors including H_2_S (Lucchesi et al., 2005), leptin, TNF-α, IL-6, and apelin may act as either vasorelaxant or constricting factors (Maenhaut and Van de Voorde, 2011). For example, leptin could induce vasodilation to modulate blood pressure homeostasis (Fruhbeck, 1999; Lembo et al., 2000); however, obesity-induced hyperleptinemia resulted in an increase of endothelin-1, which then leads to vasoconstriction (Quehenberger et al., 2002). Some reports have demonstrated that TNF-α causes vascular dilation mediated by NO (Maenhaut and Van de Voorde, 2011) or hydrogen peroxide (Cheranov and Jaggar, 2006) production. On the other hand, TNF-α can also constrict blood vessels by increasing endothelin-1 (Wort et al., 2009) and angiotensinogen levels (Brasier et al., 1996). Based on their principle functions and originating depots, these factors are summarized in Table 3.

**TABLE 3:** PVAT-derived relaxing and contracting factors.

	Relaxing factors	Contracting factors
cPVAT	Omentin Greulich et al. (2013)	Angiotensinogen
	Adrenomedullin Iacobellis et al. (2009), Silaghi et al. (2007)	Calpastatin Owen et al. (2013)
	Ang 1–7 Patel et al. (2016)
tPVAT	NO Xia et al. (2016)	Angiotensinogen, chymase, Ang I Galvez-Prieto et al. (2008)
	Prostacyclin Awata et al. (2019)	Angiotensin II (Lee et al., 2011)
	H_2_O_2_ Gao et al. (2007)	Thromboxane A_2_ Meyer et al. (2013)
	Palmitic acid methyl ester Lee et al. (2011)	
	H_2_S Fang et al., 2009, Kohn et al. (2012)	
	Leptin Fortuno et al. (2002), Lembo et al. (2000)	
	C1q/tumor necrosis factor-related protein 9 (CTRP9)Han et al. (2018)	
	Ang 1–7 Lee et al. (2009)	
aPVAT	Adiponectin, Apelin Kostopoulos et al. (2014)	Angiotensinogen Yasue et al. (2010)
	Prostacyclin Chang et al. (2012)	
mPVAT	Adiponectin Lynch et al. (2013), Weston et al. (2013), Withers et al. (2014)	Ang II Lu et al. (2010)
	NO Gil-Ortega et al. (2010), Kassan et al. (2011)	Superoxide anion Gao et al. (2006)
	H_2_O_2_ from browning mPVAT (Friederich-Persson et al., 2017)	Noradrenaline in α1 adrenoreceptor-dependent manner Ayala-Lopez et al. (2014)
	H_2_S Schleifenbaum et al. (2010)	Resistin Small et al. (2019)
	Noradrenaline via activation of β3-adrenoceptors Saxton et al. (2018)	
	Omentin Yamawaki et al. (2010)	
	Chemerin Darios et al. (2016)	

Ang, angiotensin; H_2_O_2_, Hydrogen peroxide; H_2_S, hydrogen sulfide; NO, nitric oxide.

### Perivascular Adipose Tissue-Derived Extracellular Vesicle

Inter-cell and inter-organ signaling within PVAT remain a mystery. Recently, many studies, including our own, have delved deep to identify the messengers conveying the communication between a blood vessel and its surrounding PVAT (Li et al., 2019). Apart from the secretory cytokines and chemokines factors, adipocytes also secrete many types of extracellular vesicles (EVs) (Deng et al., 2009; Ogawa et al., 2010), typically including exosomes and microvesicles. EVs play important roles in intercellular communication by selective packaging of lipids, proteins, and microRNAs (miRNAs) (Valadi et al., 2007; van Niel et al., 2018). Adipose tissue was proved to constitute an essential source of circulating exosomal miRNAs, as a form of adipokine that acts locally or distantly (Thomou et al., 2017). In addition, these EVs could be taken up by neighboring or distant cells to modulate these recipient’s functions (Bang et al., 2014; Sun et al., 2016). For instance, adipocyte-derived exosomal miRNAs enable the metabolic regulation of neighboring macrophages (Ogawa et al., 2010; Zhang et al., 2016). Vice versa, macrophages can secrete miRNA-containing exosomes to modulate local adipocyte function (Ying et al., 2017). Similarly, the endothelial-adipocyte interplay was the result of EV-mediated reciprocal trafficking of caveolin 1 (Crewe et al., 2018).

These observations led us to hypothesize that whether PVAT-adipocytes secrete exosomal miRNAs, if so, how they regulate vascular function in the context of obesity. Indeed, we have demonstrated that perivascular adipocytes produce and secrete miRNA-containing EVs, which can be taken up in neighboring VSMCs (Li et al., 2019). One of the most enriched miRNAs in PVAT and PVAT-derived EVs, miR-221-3p, is transported into adjacent VSMCs. The study further provided an EV-miR-221-3p-mediated mechanism by which PVAT triggers an early vascular remodeling in vascular inflammation (Li et al., 2019). In another study, increased miR-221/222 in the arteries promoted neointimal hyperplasia in the femoral artery following wire injury (Lightell et al., 2018).

## Conclusion

Anatomically distinct PVATs vary in developmental origin, cellular composition, and secretome. The farther away a PVAT is from the heart, the more white-like the adipocytes are. This is true even in the same stem aorta but wrapped with distinct PVATs in the chest cage and the abdominal cavity. PVAT progenitor cells include but may not be limited to mural cells (pericyte or smooth muscle cells) and fibroblasts. These potential progenitors give rise to committed preadipocytes and contribute to adipogenesis. Adipogenesis and angiogenesis appear to co-exist, and preadipocytes and pericytes may co-develop as well, which should be further studied. The developmental trajectory of PVAT adipocytes is somehow still a “bloody mess” (Rosen and Spiegelman, 2014).

Adipocytes and their neighboring vascular cells constitute a perivascular microenvironment. In an inflammatory setting, a family of intercellular message-conveying machinery is involved in these cells’ interplay. Perivascular adipocytes, partially via alteration of their secretome, modulate the nearby VSMCs (Miao and Li, 2012) and endothelial cells (Sena et al., 2017). For obesity, secretion of anti-inflammatory adiponectin is markedly reduced, whereas the generation of pro-inflammatory cytokines is dramatically elevated. Besides, the contiguity with adventitia makes it plausible that paracrine or vasocrine crosstalks between PVAT and the encircled blood vessel are reciprocal and bidirectional.

The continuing worldwide upsurge in obesity prompts us to unveil PVAT’s significant role in vascular function. A better understanding of regional heterogeneity among PVATs is just a start. PVAT’s function and dysfunction in vascular homeostasis and cardiovascular pathogeny remain our long-term tasks to pursue. Developmental fate mapping is an essential technique for answering some of these questions. Single-cell techniques have empowered this process by helping draw sophisticated cellular atlas. Some researchers have explored the heterogeneity of PVAT at a single-cell level and uncovered distinct clusters with specific signature markers and signaling pathways (Angueira et al., 2021). Creating a more precise map of such a complex tissue from single-cell sequencing data is, therefore, a challenging task, which on the other hand, opens an opportunity for us to dive into this unknown.
